# Diet and the Food Allergy Exposome Across the Lifespan: Nutritional Transitions, Microbiome–Immune Pathways, and Emerging Clinical Phenotypes

**DOI:** 10.3390/nu18213692

**Published:** 2026-11-07

**Authors:** Corina Porr, Corina Suteu, Maria Bârsan, Alina-Liliana Pintea, Dorina Maria Farcaș, Georgeta Pasca, Doina-Ileana Giurgiu

**Affiliations:** 1 Allergology Department, Faculty of Medicine, Lucian Blaga University of Sibiu, 550169 Sibiu, Romania; 2 Allergology Department, Clinical County Emergency Hospital of Sibiu, 550245 Sibiu, Romania; 3 Department of Psychiatric Neurosciences and Rehabilitation, Faculty of Medicine and Pharmacy, University of Oradea, 410073 Oradea, Romania; corina.suteu@didactic.uoradea.ro (C.S.);; 4 Department of Occupational Medicine, Iuliu Hațieganu University of Medicine and Pharmacy, 400012 Cluj-Napoca, Romania; 5 Sibiu County Emergency Clinical Hospital, 550025 Sibiu, Romania; 6 Department of Dental Medicine and Nursing, Faculty of Medicine, Lucian Blaga University of Sibiu, 550169 Sibiu, Romania; 7 Neuroscience Research Centre, Faculty of Medicine, Lucian Blaga University of Sibiu, 550169 Sibiu, Romania

**Keywords:** food allergy, exposome, nutrition, dietary patterns, gut microbiome, oral tolerance, epithelial barrier, nutritional immunology, emerging clinical phenotypes

## Abstract

Food allergy is increasingly recognized across geographic regions and age groups, although temporal trends are difficult to separate from differences in diagnostic criteria and surveillance. This narrative review examines how diet and nutrition interact with the wider exposome to influence food allergy risk, oral tolerance, and clinical phenotypes across the lifespan. We critically synthesize evidence on early-life allergen introduction, dietary diversity, fiber and microbial metabolites, Westernized and ultra-processed dietary patterns, epithelial barrier dysfunction, medication exposures, pollution, and changing food systems. Randomized trials support early peanut introduction and, with greater heterogeneity, early egg introduction as preventive approaches; by contrast, causal evidence linking most other nutritional and environmental exposures to clinically confirmed food allergy remains limited. The diet–microbiome–immune axis offers plausible pathways connecting dietary substrates and microbial metabolites to epithelial integrity and immune regulation, but human mechanistic and longitudinal evidence remains incomplete. Globalization, migration, alternative protein sources, and climate-related exposures may reshape allergen encounters. Adult-onset food allergy, pollen–food allergy syndrome, alpha-gal syndrome, and cofactor-dependent reactions further illustrate how environmental influences may alter clinical expression beyond infancy. We propose an integrated life-course framework linking nutritional and non-nutritional exposures, underlying biological pathways, and emerging allergic phenotypes, while explicitly distinguishing intervention-supported findings from observational associations and mechanistic hypotheses. Future studies should combine standardized clinical outcomes with dietary assessment, environmental exposure measurements, functional microbiome profiling, metabolomics, and immune phenotyping to identify modifiable pathways relevant to food allergy prevention.

## 1. Introduction

Food allergy represents a significant global health challenge, affecting children and adults across diverse geographic and socioeconomic settings [1,2,3]. Its epidemiology is characterized not only by variation in reported prevalence but also by changing allergen profiles, sensitization pathways, and clinical phenotypes [2,4,5,6,7,8]. Although genetic susceptibility contributes to individual risk, the relatively rapid evolution of food allergy patterns suggests an important role for environmental and lifestyle-related exposures.

The exposome, encompassing cumulative environmental, dietary, microbial, chemical, and lifestyle exposures throughout life, provides a framework for understanding these changes [9,10,11]. Within this model, food allergy may result from interactions between host susceptibility, epithelial barrier integrity, microbial ecosystems, nutritional factors, and immune regulation [9,12,13]. The timing, route, and combination of exposures may influence the balance between oral tolerance and allergic sensitization, particularly during critical periods of immune development.

Diet and nutrition occupy a central position within the food allergy exposome. Beyond providing direct exposure to allergenic proteins, dietary patterns influence microbial metabolism, epithelial homeostasis, and immune regulatory pathways [13,14,15,16,17,18,19,20]. Dietary fiber, microbial-derived short-chain fatty acids, food diversity, and processing-related changes have attracted increasing attention as potential modulators of allergic susceptibility. However, most associations involving broader dietary patterns, ultra-processed foods, and microbiome alterations remain observational or mechanistic, and their causal contribution to clinically confirmed food allergy is uncertain [14,15,16,17,18,19,20].

The strongest evidence for nutritional modification of food allergy risk comes from randomized trials demonstrating the preventive effects of early peanut introduction, with supportive but more heterogeneous findings for egg [21,22,23,24]. These results have shifted prevention strategies away from prolonged allergen avoidance toward timely, developmentally appropriate oral exposure. Nevertheless, findings concerning selected allergenic foods cannot be generalized to all nutritional or environmental interventions.

Simultaneously, urbanization, migration, climate-related environmental changes, and the diversification of global food systems are modifying patterns of dietary exposure and potentially relevant allergen sources [7,8,9,25,26,27,28,29,30,31,32,33]. The recognition of adult-onset food allergy, pollen–food allergy syndrome, alpha-gal syndrome, and cofactor-dependent reactions further illustrates the diversity of exposure-related clinical phenotypes across the lifespan [34,35,36,37,38].

Recent reviews have examined the contribution of environmental exposures to food allergy development, including microbial influences, pollutants, and synthetic chemicals [39]. Building on this evidence, the present review offers a distinct nutrition-centered, life-course synthesis that connects dietary transitions with epithelial barrier function, microbiome–immune interactions, and changing clinical phenotypes. Its added value lies in three complementary aspects: (i) comparing intervention-supported nutritional prevention strategies with predominantly observational dietary and environmental associations; (ii) integrating mechanistic pathways of oral tolerance and allergic sensitization with the emergence of different food allergy phenotypes; and (iii) examining how nutritional exposures and clinical management priorities differ from pregnancy and infancy to childhood, adolescence, and adulthood. Rather than treating nutritional determinants and environmental influences as independent factors, this framework emphasizes their interactions, the timing and route of exposure, and the distinction between mechanistic plausibility and clinically demonstrated effects. By integrating these dimensions, the review aims to identify evidence gaps and clinically relevant priorities for future research.

### Review Approach and Added Value

This article presents a narrative, non-systematic review examining the interactions between nutrition, environmental exposures, immune regulation, and food allergy across the lifespan. Its purpose is to integrate clinical, epidemiological, and mechanistic evidence within a nutrition-centered exposome framework rather than to provide an exhaustive or quantitatively pooled assessment of the literature.

Relevant publications were identified through targeted literature searches and examination of reference lists from pertinent articles and clinical guidelines. Studies were selected based on their relevance to the review’s main themes, with particular attention to study design, clinical applicability, and the strength of the available evidence.

The literature discussed encompasses international clinical guidelines, randomized controlled trials, longitudinal and cross-sectional observational studies, experimental investigations, and relevant reviews. Evidence was considered in relation to its relevance to the major themes of the manuscript, methodological strengths and limitations, and clinical applicability. Particular emphasis was placed on studies reporting clinically confirmed food allergy and randomized preventive interventions. Findings concerning allergic sensitization, immune biomarkers, microbial composition, and experimental models were interpreted separately from evidence demonstrating clinically established food allergy.

No registered review protocol, exhaustive systematic search, duplicate study screening, formal risk-of-bias assessment, or meta-analysis was undertaken. Accordingly, this review does not claim comprehensive evidence identification or systematic evidence grading. Its objective is to provide a critical, clinically oriented synthesis that identifies established findings, unresolved uncertainties, and priorities for future research.

## 2. Nutritional and Environmental Exposome of Food Allergy

Food allergy develops within a complex network of nutritional, environmental, microbial, and host-related influences that vary across populations and throughout life. The exposome framework provides an integrated approach to understanding these interactions by considering cumulative exposures, their timing, and their potential biological consequences [9,10,11]. Rather than attributing allergic disease to individual risk factors, this perspective emphasizes how combinations of exposures may influence epithelial integrity, immune regulation, and the balance between sensitization and oral tolerance [9,12,13].

### 2.1. Changing Dietary Patterns, Urbanization, and Migration

The epidemiology of food allergy exhibits substantial geographic heterogeneity, reflecting differences in dietary practices, environmental exposures, population characteristics, and diagnostic methodology [2,4,5,6,7,40,41]. Although North America, Western Europe, and Australia have historically reported a substantial burden of food allergy, growing recognition in rapidly urbanizing regions suggests that its distribution is increasingly global [2,5,42,43,44,45,46].

These differences should not be interpreted solely as variations in disease prevalence. Regional food consumption, aeroallergen exposure, and access to specialist diagnosis may influence both recognized allergen profiles and clinical phenotypes [7,25,34,42,43,44,45,46].

Urbanization is frequently accompanied by nutritional transitions involving greater consumption of processed foods, changes in dietary diversity, and reduced intake of selected fiber-rich foods [2,9,19,20]. Such transitions occur alongside changes in microbial exposure, biodiversity, pollution, housing conditions, and medication use. Consequently, urbanization represents a multidimensional exposure rather than an independent biological determinant [9,12,45].

Migration provides another informative model of environmental change. Relocation between geographic regions may modify dietary habits, food allergen exposure, microbial environments, and aeroallergen sensitization patterns [2,5,9]. Differences observed between migrant generations suggest that environmental conditions may contribute to food allergy expression. However, confounding by socioeconomic circumstances, healthcare access, and diagnostic practices limits causal interpretation.

Importantly, neither Westernized dietary patterns nor urbanization has been established as an independent cause of clinically confirmed food allergy. Their relevance lies in the potential interaction of multiple nutritional and environmental determinants during different periods of susceptibility.

### 2.2. Epithelial Barrier Dysfunction and Cellular and Molecular Mechanisms of Food Allergic Sensitization

The development of IgE-mediated food allergy involves a complex interaction between epithelial barrier dysfunction, innate immune activation, antigen presentation, type 2 adaptive immune responses, and allergen-specific IgE production. These processes are influenced by host susceptibility, environmental exposures, microbial signals, and the route and timing of allergen encounter. Although several molecular pathways have been identified in experimental models, their relative contribution to clinically confirmed food allergy in humans remains incompletely understood [12,13,47].

#### 2.2.1. Epithelial Barrier Dysfunction and Initiation of Type 2 Inflammation

The skin and gastrointestinal epithelium constitute essential interfaces between environmental allergens and the immune system. Impaired epithelial integrity may facilitate allergen penetration and promote inflammatory signals that favor allergic sensitization. In infants with atopic dermatitis, epidermal barrier dysfunction, including defects involving filaggrin and other structural proteins, is associated with an increased risk of food allergy [12,47,48].

Following epithelial injury or inflammatory stimulation, keratinocytes and other epithelial cells can release the alarmins thymic stromal lymphopoietin (TSLP), interleukin-25 (IL-25), and interleukin-33 (IL-33). These mediators contribute to the development of a type 2 inflammatory microenvironment by influencing dendritic cells, type 2 innate lymphoid cells (ILC2s), and other immune populations [13,47].

TSLP can promote the activation and functional polarization of dendritic cells, including increased expression of OX40 ligand (OX40L), thereby facilitating the differentiation of naïve CD4+ T cells toward a T-helper 2 (Th2) phenotype. IL-33 signals through the ST2 receptor and can amplify type 2 inflammatory responses, while IL-25 contributes to ILC2 activation and cytokine production. These pathways provide mechanistic links between epithelial barrier dysfunction and the initiation or amplification of allergen-specific immune responses [13,47].

#### 2.2.2. Antigen Presentation, Th2 Polarization, and IgE Class Switching

Following allergen uptake, activated antigen-presenting cells process dietary proteins and present allergen-derived peptides to naïve CD4+ T lymphocytes. In an inflammatory environment enriched in type 2-promoting signals, antigen presentation may favor Th2 differentiation through pathways involving OX40–OX40L interactions and IL-4-dependent activation of signal transducer and activator of transcription 6 (STAT6) [13,47].

Activated Th2 lymphocytes produce cytokines including IL-4, IL-5, and IL-13. IL-4 and IL-13 contribute to allergic inflammation and promote immunoglobulin class switching in B lymphocytes, particularly through IL-4 receptor alpha (IL-4Rα)-associated signaling. In cooperation with CD40–CD40L interactions and T-cell help, these signals facilitate the production of allergen-specific immunoglobulin E (IgE) [13,47].

IL-5 contributes primarily to eosinophil development, recruitment, and activation, whereas IL-13 also influences epithelial function, mucus production, and tissue inflammatory responses. T follicular helper cell populations may additionally support germinal-center B-cell responses and allergen-specific antibody production [13].

The resulting allergen-specific IgE binds to the high-affinity Fc epsilon receptor I (FcεRI) expressed on mast cells and basophils. This process establishes cellular sensitization but does not necessarily indicate clinically manifest food allergy. The presence of allergen-specific IgE alone is therefore insufficient to establish clinical reactivity [13,47].

#### 2.2.3. Mast Cells, Basophils, and the Effector Phase of Food Allergy

In sensitized individuals, subsequent exposure to the relevant food allergen may induce cross-linking of FcεRI-bound IgE on mast cells and basophils, initiating intracellular signaling cascades involving kinases such as Lyn and Syk. These pathways promote calcium-dependent cellular activation, degranulation, and the generation of inflammatory mediators [13,47]. Mast-cell activation results in the release of preformed mediators, including histamine and tryptase, followed by the synthesis of lipid mediators such as leukotrienes and prostaglandins. Basophils also contribute to immediate hypersensitivity responses and may amplify type 2 inflammation through mediator and cytokine release [13,47].

These mediators can produce cutaneous, gastrointestinal, respiratory, and cardiovascular manifestations, ranging from localized symptoms to systemic anaphylaxis. However, clinical severity depends on multiple factors, including allergen dose, individual reaction thresholds, tissue distribution of effector cells, and the presence of relevant cofactors [13,47].

The contribution of mast cells and basophils emphasizes the distinction between allergic sensitization and clinically expressed disease. Although IgE-mediated effector activation is central to immediate food-allergic reactions, sensitization biomarkers do not reliably predict reaction severity [13].

#### 2.2.4. Microbiome–Epithelial–Immune Interactions and Potential Molecular Targets

The intestinal microbiome may influence allergic susceptibility through interactions with epithelial cells, antigen-presenting cells, and adaptive immune populations. Microbial metabolites, particularly short-chain fatty acids derived from dietary fiber fermentation, can modulate epithelial barrier function and immune regulatory pathways [14,15,16,17,18].

Experimental evidence suggests that microbial metabolites may influence regulatory T-cell differentiation, inflammatory signaling, and the balance between tolerogenic and type 2 immune responses. Conversely, alterations in microbial communities and metabolic activity may contribute to an inflammatory environment that facilitates allergic sensitization. However, reproducible causal relationships between specific microbial alterations and clinically confirmed food allergy in humans have not been established [14,15,16,17,18].

Several molecular pathways implicated in food allergy have therefore emerged as potential targets for therapeutic investigation. These include epithelial-derived alarmin pathways involving TSLP, IL-25, and IL-33; type 2 cytokine signaling through IL-4 and IL-13; IgE–FcεRI interactions; and intracellular signaling pathways associated with mast-cell and basophil activation [13,47].

Targeting these pathways may attenuate allergic inflammation or reduce effector-cell responsiveness. Nevertheless, therapeutic modification of established allergic responses should be distinguished from prevention of initial sensitization, and clinical efficacy for one objective cannot automatically be extrapolated to the other [13,47].

Furthermore, interventions targeting immune pathways must be evaluated in relation to their safety, developmental timing, and potential effects on normal immune regulation.

#### 2.2.5. Integration Within the Food Allergy Exposome

These mechanisms illustrate how nutritional, microbial, and environmental exposures may interact with host susceptibility to influence the balance between oral tolerance and allergic sensitization [9,12,13,14,15,16,17,18].

Epithelial barrier dysfunction may facilitate allergen entry and initiate type 2 immune responses, whereas appropriate gastrointestinal antigen exposure and immunoregulatory signals may favor tolerance. The clinical consequences depend on the interplay between exposure route, antigen characteristics, immune maturation, microbial activity, and genetic or acquired susceptibility [12,13,47].

Importantly, the mechanistic pathways described above primarily concern IgE-mediated food allergy. They should not be assumed to explain predominantly non-IgE-mediated disorders, including food protein-induced enterocolitis syndrome, which involve partially distinct and incompletely characterized immune mechanisms [13].

Although these pathways provide biologically plausible targets for future interventions, evidence supporting their modification as a strategy for primary food allergy prevention remains substantially less developed than evidence supporting early oral introduction of selected allergens [21,22,23,24,47].

An integrated exposome framework should therefore distinguish experimentally demonstrated mechanisms, human observational associations, and interventions supported by randomized clinical outcomes.

### 2.3. Microbial, Chemical, and Lifestyle Exposures

The intestinal microbiome represents a major biological interface between diet and immune regulation. Microbial communities and their metabolites contribute to epithelial homeostasis and regulatory immune processes, while nutritional composition influences microbial metabolic activity [14,15,16,17,18].

Environmental microbial exposure may be modified by delivery mode, antibiotic treatment, housing conditions, and contact with natural environments [9,14,15]. Although differences in microbial composition have been associated with food allergy, no universally reproducible microbial signature has been established for clinical prediction [14,15,16].

Antibiotic exposure during early life has also been associated with subsequent allergic outcomes. However, confounding by infection burden, healthcare utilization, and underlying susceptibility limits conclusions regarding an independent causal effect [9,14,15].

Environmental pollution constitutes another potential component of the food allergy exposome. Airborne pollutants, tobacco smoke, pesticides, and selected household chemicals may influence epithelial integrity and immune responses through oxidative stress and inflammatory mechanisms [12,26,27,28].

Nevertheless, evidence directly linking these exposures to clinically confirmed food allergy remains substantially less consistent than evidence concerning respiratory allergic diseases. Studies frequently investigate allergic sensitization or broader atopic outcomes rather than challenge-confirmed food allergy.

The combined influence of nutritional, microbial, chemical, and lifestyle exposures therefore remains biologically plausible but incompletely established in humans.

### 2.4. Exposure Timing and Converging Biological Pathways

The biological consequences of exposome-related factors may depend on exposure timing, duration, route, and host susceptibility. Pregnancy, infancy, and early childhood represent important developmental periods during which epithelial barriers, microbial ecosystems, and immune regulatory pathways undergo maturation [9,12,13].

However, environmental influences are not restricted to early life. The recognition of adult-onset food allergy and environmentally acquired phenotypes suggests that changing exposures may also contribute to allergic sensitization or modify clinical expression in adulthood [35,37,38].

Different exposures may converge on interconnected biological pathways involving epithelial barrier function, microbial metabolism, and immune regulation. Dietary composition may alter microbial metabolic activity, while pollution or medication-related exposures may affect epithelial and microbial environments [9,10,11,12,14,15,16,17,18].

The strength of supporting evidence nevertheless varies considerably. Randomized trials establish that early introduction of selected allergenic foods can modify food allergy risk, whereas most other proposed exposome determinants remain supported predominantly by observational associations or experimental mechanisms [14,15,16,17,18,19,20,21,22,23,24,26,27,28].

An integrated exposome model should therefore distinguish established causal interventions from potential risk modifiers and biologically plausible hypotheses.

**Figure 1:**
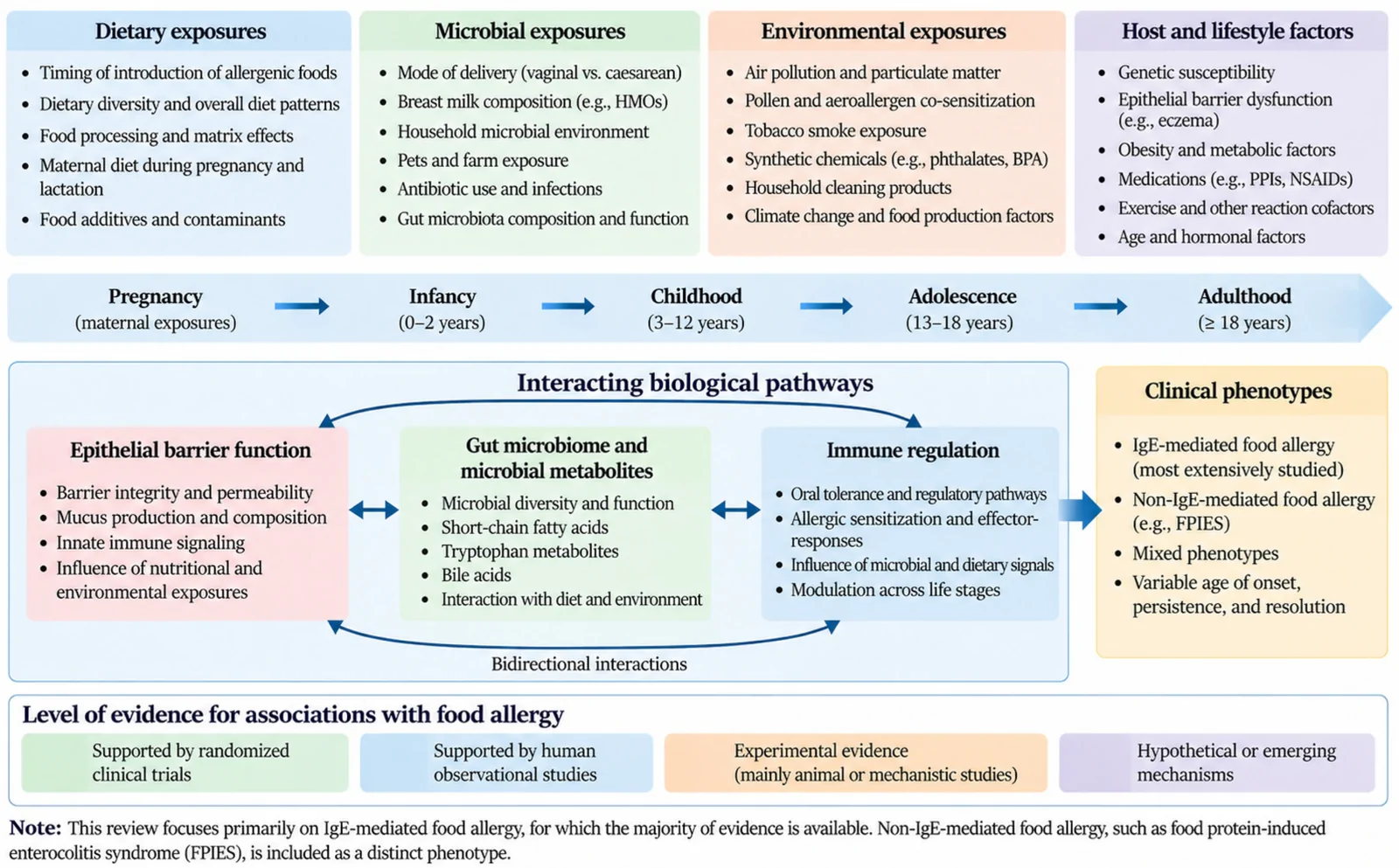
Nutritional and environmental determinants of food allergy across the lifespan. The diagram illustrates proposed interactions between dietary, microbial, environmental, and host-related exposures, epithelial barrier integrity, gut microbiome activity, and immune regulation. Horizontal arrows indicate progression across life stages, while bidirectional arrows represent interconnected biological processes rather than established causal relationships. The different background colors distinguish exposure categories, biological pathways, and clinical phenotypes. In the evidence-level legend, green indicates evidence from randomized clinical trials, blue indicates human observational evidence, orange indicates experimental evidence, and purple indicates hypothetical or emerging mechanisms. These evidence categories should not be interpreted as demonstrating causality for every depicted association.

Figure 1 summarizes the principal nutritional and environmental exposures, their proposed interactions with epithelial, microbial, and immune pathways, and their potential contribution to clinically relevant food allergy.

## 3. Early-Life Nutrition and Food Allergy Prevention

Early life represents a critical developmental period during which nutritional exposures, epithelial barrier maturation, intestinal microbial development, and immune regulation interact to influence the establishment of oral tolerance [9,12,13]. Although genetic susceptibility contributes to food allergy risk, randomized controlled trials have demonstrated that the timing and route of exposure to selected dietary allergens can modify disease development [21,22,23,24].

Within the nutritional exposome, early feeding practices should therefore be considered alongside epithelial integrity, microbial maturation, and environmental conditions. However, the strength of evidence differs considerably among proposed preventive interventions, and findings concerning specific allergenic foods should not be generalized to all dietary exposures.

### 3.1. The Dual-Allergen Exposure Hypothesis and Molecular Mechanisms of Oral Tolerance

Oral tolerance is an active, antigen-specific immunological process that enables the immune system to recognize dietary proteins without generating harmful inflammatory responses. Its establishment depends on coordinated interactions among intestinal epithelial cells, antigen-presenting cells, regulatory lymphocytes, microbial metabolites, and local immunoregulatory signals. Disruption of these mechanisms may favor allergic sensitization, particularly during early immune development [13,49,50].

Following ingestion, dietary antigens cross the intestinal epithelial barrier through regulated pathways involving specialized epithelial cells, including microfold (M) cells, and other antigen-sampling mechanisms within the intestinal mucosa. Antigens are subsequently captured and processed by antigen-presenting cells, including intestinal dendritic cells. Under non-inflammatory conditions, tolerogenic antigen-presenting-cell populations promote regulatory rather than inflammatory immune responses [49,50].

A central mechanism involves the presentation of dietary antigens to naïve CD4+ T lymphocytes in intestinal draining lymph nodes. Retinoic acid and transforming growth factor-beta (TGF-β) contribute to the differentiation of antigen-specific forkhead box P3-positive (FOXP3+) peripheral regulatory T cells (pTregs). These cells subsequently migrate toward intestinal tissues through mechanisms involving chemokine receptor CCR9 and integrin α4β7, where they contribute to the maintenance of mucosal immune homeostasis [49,50].

Regulatory T cells suppress excessive immune activation through several complementary pathways, including the production of interleukin-10 (IL-10) and TGF-β, inhibition of inflammatory T-cell responses, and regulation of antigen-presenting-cell function. These processes help limit inappropriate type 2 immune responses against dietary proteins and contribute to antigen-specific immune tolerance. Recent experimental investigations have additionally identified RORγt-expressing antigen-presenting-cell populations involved in the induction of food-specific regulatory T cells, indicating that the cellular regulation of oral tolerance extends beyond conventional dendritic-cell pathways. These findings remain predominantly experimental and require further validation in humans [49,50,51].

The intestinal microbiome contributes to these immunoregulatory processes through interactions with epithelial cells, antigen-presenting cells, and regulatory lymphocytes. Microbial fermentation of dietary fiber generates short-chain fatty acids, particularly acetate, propionate, and butyrate, which may influence regulatory T-cell differentiation, epithelial barrier integrity, and inflammatory signaling. Experimental evidence supports a functional relationship between microbial metabolites and oral tolerance. However, the extent to which individual microbial pathways independently determine clinically confirmed food allergy risk in humans remains incompletely established [15,16,17,18].

The dual-allergen exposure hypothesis provides a complementary explanation for the development of allergic sensitization. Exposure to food proteins through an inflamed or impaired skin barrier, particularly in infants with atopic dermatitis, may favor type 2 inflammatory responses and allergen-specific IgE production. In contrast, appropriately timed gastrointestinal exposure may support the establishment of oral tolerance. The immunological consequences of allergen exposure therefore depend not only on antigen characteristics but also on the exposure route, epithelial integrity, inflammatory environment, and developmental timing [12,13,47,49,50,52,53].

These mechanisms have important implications for food allergy prevention. Randomized clinical trials demonstrate that early oral introduction of selected allergenic foods, particularly peanut, can reduce subsequent food allergy development in defined infant populations. Nevertheless, clinical efficacy does not establish that every proposed cellular pathway is necessary for the observed protective effect. Many detailed mechanisms of oral tolerance have been characterized predominantly in experimental models, and their relative contributions in humans require further investigation [21,22,23,24,49,50].

From a preventive perspective, maintaining epithelial barrier integrity, supporting appropriate microbial–immune interactions, and facilitating developmentally appropriate oral allergen exposure represent biologically relevant strategies. However, only interventions supported by sufficiently robust clinical evidence should be considered established preventive recommendations. Improved understanding of antigen presentation, regulatory T-cell differentiation, and microbiome-derived immunomodulatory signals may help identify future molecular targets for individualized food allergy prevention [18,21,22,23,24,49,50].

### 3.2. Evidence from Randomized Prevention Trials

The Learning Early About Peanut Allergy (LEAP) trial established that regular peanut consumption beginning during infancy substantially reduced peanut allergy at 60 months among children at increased allergic risk [21]. These findings provided direct evidence that early nutritional exposure can modify the development of allergen-specific immune responses.

The long-term LEAP-Trio follow-up further demonstrated persistence of this protective effect into adolescence. At approximately 12 years of age, peanut allergy was identified in 4.4% of participants originally assigned to peanut consumption compared with 15.4% of those assigned to avoidance [54]. These results support a durable preventive effect of early peanut introduction, although the optimal exposure patterns required for sustained protection remain an area of investigation.

Evidence concerning other allergenic foods is more heterogeneous. The Enquiring About Tolerance (EAT) trial evaluated the early introduction of six allergenic foods in breastfed infants. Although the primary intention-to-treat analysis did not demonstrate a statistically significant reduction in food allergy, per-protocol analyses suggested benefits, particularly for peanut and egg [22]. Difficulties with adherence and the potential for selection bias in per-protocol analyses limit interpretation.

The Prevention of Egg Allergy with Tiny Amount Intake (PETIT) trial demonstrated that stepwise introduction of heated egg reduced egg allergy at 12 months in infants with eczema receiving active dermatological treatment [23]. These findings support the importance of allergen preparation and clinical context when evaluating nutritional interventions.

The PreventADALL trial investigated early introduction of peanut, cow’s milk, wheat, and egg in a general infant population. The food intervention reduced food allergy at 36 months, whereas the evaluated skin emollient intervention did not demonstrate comparable protection [55]. However, because the feeding intervention began at 3 months, its protocol should not be interpreted as a general recommendation to introduce complementary foods before 4 months.

Collectively, these studies establish early oral allergen exposure as a modifiable determinant of food allergy risk. Nevertheless, differences in study populations, intervention timing, allergen preparation, adherence, and diagnostic outcomes limit direct comparison and extrapolation to other foods [21,22,23,24,54,55]. The principal methodological characteristics and outcomes of the LEAP, EAT, PETIT, and PreventADALL trials are compared in Table 1.

### 3.3. Complementary Feeding, Dietary Diversity, and Prevention Recommendations

International prevention recommendations increasingly support introducing selected allergenic foods during developmentally appropriate complementary feeding rather than delaying their introduction solely to prevent allergy [56,57].

The EAACI prevention guideline recommends introducing well-cooked egg during complementary feeding and suggests age-appropriate peanut introduction in populations with a high prevalence of peanut allergy [56]. In infants with severe eczema, known food allergy, or suspected sensitization, individualized evaluation may be required before introducing specific allergens.

Once tolerated, allergenic foods should generally remain part of the diet. However, the optimal frequency and quantity required for maintaining tolerance have not been established for all foods and populations.

Beyond individual allergens, dietary diversity may influence immune development through broader antigen exposure and the provision of substrates for microbial metabolism. Observational studies have associated greater diversity during complementary feeding with more favorable allergic outcomes [19].

Nevertheless, dietary diversity is influenced by nutritional quality, food preparation, socioeconomic conditions, and feeding practices. Its independent causal contribution to food allergy prevention therefore remains uncertain.

Recent EAACI guidance emphasizes nutritionally adequate complementary feeding, including a variety of nutrient-dense foods and developmentally appropriate allergen introduction [58]. These recommendations support integrating allergy prevention into overall healthy infant nutrition rather than treating allergen exposure as an isolated intervention.

### 3.4. Maternal Nutrition, Breastfeeding, and Microbiome-Targeted Interventions

Maternal diet, breastfeeding, and early microbial exposures are additional components of the nutritional exposome.

Current evidence does not demonstrate that maternal avoidance of common food allergens during pregnancy or breastfeeding prevents food allergy in offspring. Routine maternal dietary restriction for this purpose is therefore not recommended [56,59].

Breastfeeding should be encouraged because of its established nutritional and developmental benefits, although its independent protective effect against clinically confirmed food allergy remains uncertain [56,59].

The infant intestinal microbiome also contributes to epithelial maturation and regulatory immune processes. Experimental findings suggest that microbial communities and their metabolites may influence oral tolerance, but no universal microbial signature has been established for predicting food allergy [14,15,16,17,18].

Despite growing interest in probiotics, prebiotics, synbiotics, vitamin D, omega-3 fatty acids, and other supplements, available evidence remains insufficient to recommend their routine administration specifically for primary food allergy prevention [56,57,60].

Similarly, associations between cesarean delivery, early antibiotic exposure, microbial disruption, and subsequent allergic outcomes remain vulnerable to confounding and should not be interpreted as established causal relationships [9,14,15].

### 3.5. Clinical Implications and Remaining Uncertainties

Early nutritional prevention provides one of the clearest examples of how modifying a component of the exposome can influence food allergy development.

The strongest evidence supports timely peanut introduction, with additional but more heterogeneous evidence for egg and selected multi-allergen introduction strategies [21,22,23,24,54,55,56,57].

By contrast, the preventive effects of broader dietary diversity, microbiome-targeted supplementation, maternal dietary modification, and other nutritional interventions remain incompletely established.

Future studies should determine how allergen exposure interacts with dietary composition, epithelial integrity, microbial function, and host susceptibility during specific developmental windows. Particular attention should be given to clinically confirmed outcomes, intervention adherence, long-term tolerance, and the generalizability of prevention strategies across different populations.

An integrated nutritional-exposome approach may ultimately support more individualized prevention strategies while avoiding recommendations that extend beyond the available clinical evidence [61].

## 4. Diet–Microbiome–Immune Interactions in Food Allergy

The diet–microbiome–immune axis represents a central component of the food allergy exposome, providing a mechanistic framework linking nutritional exposures to epithelial homeostasis, immune regulation, and oral tolerance [13,14,15,16,17,18]. Dietary components act not only as potential allergens but also as substrates for microbial metabolism, influencing the production of bioactive compounds and the intestinal environment in which immune responses develop.

Experimental studies demonstrate that microbial communities and their metabolites can modulate pathways involved in allergic sensitization [15,16,18]. However, human evidence remains heterogeneous, and the extent to which specific dietary patterns or microbiome alterations causally influence clinically confirmed food allergy is uncertain. Distinguishing mechanistic findings from observational associations and intervention-supported outcomes is therefore essential.

### 4.1. Dietary Fiber, Microbial Metabolites, and Immune Regulation

Dietary fiber is an important link between nutrition and intestinal microbial function. Fermentation of non-digestible carbohydrates by gut microorganisms generates short-chain fatty acids (SCFAs), particularly acetate, propionate, and butyrate, which contribute to epithelial integrity and immune homeostasis [17,18].

SCFAs influence several regulatory pathways, including epithelial barrier function, inflammatory signaling, and regulatory T-cell responses. Experimental evidence indicates that these metabolites may promote tolerogenic immune environments and reduce susceptibility to food allergen sensitization [17,18].

In a murine model, Tan et al. demonstrated that high-fiber feeding and short-chain fatty acids enhanced oral tolerance and reduced food-allergic responses through mechanisms involving CD103+ dendritic cells, regulatory immune pathways, and SCFA receptors [18]. The evaluated outcomes included allergic responses and immune parameters in animals, rather than clinically confirmed food allergy prevention in humans.

Human observational studies have reported associations between intestinal microbial profiles, metabolic activity, and allergic outcomes. However, these findings are heterogeneous and do not establish that increasing dietary fiber or specific SCFAs prevents challenge-confirmed food allergy in humans [14,15,16,17,18,62].

Beyond SCFAs, microbial metabolites derived from tryptophan and bile acids may influence epithelial and immune regulation. Tryptophan-derived indoles have been investigated in experimental models involving aryl hydrocarbon receptor signaling, whereas bile acid-related pathways have been linked to intestinal immune regulation. Nevertheless, much of this evidence concerns molecular signaling, immune biomarkers, or experimental allergic responses rather than clinically confirmed food allergy prevention. Direct human interventional evidence remains insufficient [14,15,16,17,18,62].

These findings highlight the importance of distinguishing biological plausibility from clinical efficacy. Future studies should evaluate functional microbial metabolites alongside standardized clinical outcomes, including oral food challenge-confirmed allergy, rather than relying exclusively on microbial composition or immunological biomarkers.

### 4.2. Microbiome Diversity and Functional Dysbiosis

Human observational studies have reported differences in intestinal microbial composition between food-allergic and non-allergic individuals, particularly during infancy and early childhood [14,15,16]. However, these associations vary across populations and study designs, and no reproducible microbial signature has been validated for predicting clinically confirmed food allergy.

Experimental studies have demonstrated that selected microbial communities and their metabolites can influence epithelial barrier function, regulatory immune responses, and susceptibility to allergic sensitization [15,16,18]. These findings provide mechanistic evidence but cannot be directly extrapolated to human disease prevention.

An important limitation of human microbiome research is the heterogeneity of clinical endpoints. Some studies evaluate microbial composition or immune biomarkers, whereas others assess allergen sensitization or clinically diagnosed food allergy. These outcomes are not interchangeable, and associations with immunological markers do not necessarily establish clinical allergic disease.

Microbial function may also be more informative than taxonomic diversity alone. Functional metagenomic and metabolomic approaches can characterize pathways related to short-chain fatty acid production, tryptophan metabolism, and bile acid transformation [14,15,16,17,18,62]. However, evidence linking these metabolic pathways to food allergy remains predominantly experimental or observational, and their independent causal contribution to clinically confirmed disease has not been established.

Future longitudinal studies should combine dietary assessment, functional microbiome profiling, and immunological measurements with standardized clinical outcomes, preferably including oral food challenges, to determine whether microbial signatures or metabolites have clinically meaningful predictive or preventive value.

### 4.3. Dietary Diversity, Nutritional Quality, and Oral Tolerance

Observational studies have associated greater dietary diversity during infancy with more favorable allergic outcomes [19]. A varied diet may support microbial metabolic diversity and provide fiber, micronutrients, and bioactive compounds involved in intestinal homeostasis.

However, dietary diversity is a heterogeneous exposure influenced by food quality, preparation methods, consumption frequency, developmental timing, and socioeconomic factors.

Its potential protective effects must also be distinguished from the established benefits of early introduction of selected allergenic foods, which have been demonstrated in randomized trials [21,22,23,24].

Current evidence therefore supports dietary diversity as an important component of healthy nutrition, but not as an independently validated intervention for preventing food allergy.

### 4.4. Westernized Diets and Ultra-Processed Foods

Westernized dietary patterns, frequently characterized by lower fiber intake and greater consumption of refined and highly processed foods, may influence microbial metabolism, epithelial integrity, and inflammatory responses [17,18,19,20].

Ultra-processed foods have attracted particular interest because of their nutritional composition, processing-related modifications, and potential effects of selected additives on intestinal and immune function.

An EAACI task force report highlighted emerging associations between ultra-processed food consumption and allergic outcomes in children [20]. Nevertheless, the available evidence remains predominantly observational, and a direct causal relationship with clinically confirmed food allergy has not been established.

An important methodological limitation is the difficulty of distinguishing food processing from overall dietary quality, socioeconomic conditions, obesity-related factors, and other lifestyle exposures. Furthermore, studies frequently assess sensitization or broad allergic outcomes rather than challenge-confirmed food allergy.

Food processing may also modify protein structure, digestibility, and IgE-binding properties, but these effects are allergen-specific and cannot be generalized across food categories [31,63].

Dietary processing remains a relevant component of the nutritional exposome and warrants further investigation using standardized dietary and clinical assessments.

### 4.5. Microbiome-Directed Nutritional Interventions: Prebiotics, Probiotics, Postbiotics, and Synbiotics

The recognition of the gut microbiome as a regulator of mucosal immune homeostasis has stimulated interest in microbiome-directed nutritional interventions for food allergy prevention. Prebiotics, probiotics, postbiotics, and synbiotics represent conceptually distinct approaches that may influence microbial metabolic activity, epithelial barrier function, and immunological pathways involved in allergic sensitization and oral tolerance. However, their potential preventive effects require cautious interpretation, as mechanistic plausibility has not consistently translated into demonstrated clinical protection against food allergy [15,16,17,18,56,57].

Prebiotics are substrates selectively utilized by host microorganisms that confer a health benefit. Certain non-digestible carbohydrates, including selected oligosaccharides, can influence microbial metabolic activity and the abundance of specific microbial populations. Their fermentation may promote the generation of short-chain fatty acids (SCFAs), particularly acetate, propionate, and butyrate. These metabolites can participate in epithelial barrier maintenance and immune regulation through mechanisms involving G-protein-coupled receptors, histone deacetylase inhibition, and regulatory T-cell differentiation. Nevertheless, human studies have not established that routine prebiotic supplementation reliably prevents clinically confirmed food allergy [17,18,56,64].

Probiotics are live microorganisms that, when administered in adequate amounts, confer a health benefit on the host. Experimental investigations suggest that selected probiotic strains may influence intestinal epithelial integrity, dendritic-cell function, regulatory T-cell responses, and the balance between type 2 inflammatory and tolerogenic immune pathways. Potential mechanisms include modulation of IL-10 and TGF-β signaling, changes in microbial metabolites, and attenuation of epithelial inflammatory responses. However, these effects are strain-specific and context-dependent. Clinical studies remain heterogeneous regarding microbial strains, doses, duration, intervention timing, baseline microbiome characteristics, and diagnostic endpoints. Consequently, evidence from one probiotic formulation cannot be generalized to other strains or combinations [15,16,56,57,65].

Postbiotics consist of preparations of inanimate microorganisms and/or their components that confer a health benefit. They may influence host–microbial interactions without requiring the administration of viable organisms. Experimental studies suggest potential effects on epithelial signaling, innate immune recognition, and immunoregulatory pathways. Importantly, purified microbial metabolites, including isolated SCFAs, should not automatically be classified as postbiotics under the current consensus definition. Evidence supporting postbiotic preparations specifically for primary food allergy prevention remains insufficient, and clinical trials using standardized preparations and confirmed allergic outcomes are needed [66].

Synbiotics comprise mixtures of live microorganisms and substrates selectively utilized by host microorganisms that confer a health benefit. Current consensus terminology distinguishes complementary synbiotics from synergistic synbiotics. Their proposed rationale involves interactions between administered microorganisms, microbial substrates, and host microbial ecosystems. Depending on the formulation, synbiotics may influence SCFA production, epithelial barrier function, and immune regulatory responses. Nevertheless, their biological effects depend on the specific microbial strains and substrates used, and clinical evidence demonstrating a consistent preventive benefit against food allergy remains limited [56,67].

From a mechanistic perspective, these interventions may converge on interconnected pathways, including modulation of intestinal microbial metabolites, enhancement of epithelial barrier function, regulation of antigen-presenting cells, expansion or functional modulation of regulatory T-cell populations, and attenuation of type 2 inflammatory responses. However, changes in microbial diversity, cytokine profiles, allergen-specific IgE concentrations, or other immunological biomarkers should not be considered equivalent to prevention of clinically confirmed food allergy. Experimental and observational findings must therefore be distinguished from randomized clinical evidence demonstrating a reduction in disease incidence [15,16,17,18,56,57].

Current EAACI prevention guidance provides no recommendation for or against the use of prebiotics, probiotics, or synbiotics during pregnancy, breastfeeding, or infancy specifically to prevent food allergy. This position reflects insufficient and heterogeneous evidence rather than proof that these interventions are ineffective. For postbiotics, evidence directly addressing primary food allergy prevention remains particularly limited. Accordingly, none of these approaches can currently be presented as an established universal preventive strategy [56,57,66].

An important distinction must also be maintained between primary prevention and treatment of established food allergy. Studies examining microbiome-directed interventions as adjuncts to allergen immunotherapy address therapeutic objectives, such as desensitization or sustained unresponsiveness, and cannot independently establish efficacy for primary prevention in previously non-allergic individuals. Similarly, improvements in immune biomarkers or gastrointestinal microbial profiles do not necessarily correspond to clinically meaningful changes in reaction thresholds or long-term outcomes.

Future randomized controlled trials should employ well-characterized formulations, standardized intervention protocols, appropriate longitudinal follow-up, and clinically meaningful outcomes, preferably including oral food challenge-confirmed allergy. Integration of functional microbiome profiling, metabolomics, and immunological phenotyping may further clarify which biological effects are reproducible and whether they translate into clinically relevant protection [15,16,17,18,56,57].

### 4.6. Clinical Interpretation and Research Implications

The diet–microbiome–immune axis offers a biologically plausible explanation for interactions between nutritional exposures, microbial metabolism, epithelial integrity, and immune regulation. However, mechanistic plausibility does not necessarily translate into clinically effective dietary interventions.

Among nutritional approaches, early oral introduction of selected allergens has the strongest causal evidence for food allergy prevention. In contrast, the proposed effects of dietary fiber, broader dietary patterns, microbial metabolites, and microbiome-targeted supplementation remain incompletely established in humans.

Future investigations should integrate repeated dietary assessment with functional microbiome profiling, metabolomics, epithelial biomarkers, and standardized clinical outcomes. Particular attention should be given to exposure timing, individual susceptibility, and the distinction between sensitization and clinically confirmed disease.

Such studies may help identify whether nutritional modification of microbial pathways can produce measurable and clinically relevant effects on food allergy risk.

Table 2 summarizes the principal nutritional determinants, their proposed biological mechanisms, and the current strength of evidence.

## 5. Changing Food Systems and Emerging Allergenic Exposures

Global food systems are undergoing substantial transformations driven by urbanization, international trade, changing dietary preferences, technological innovation, and sustainability concerns. These developments are modifying the diversity and characteristics of dietary proteins encountered across populations [7,8,29,30,31,32,33]. Within the food allergy exposome, such changes may influence allergen exposure and sensitization patterns, although increased consumption of unfamiliar foods does not necessarily translate into increased clinically confirmed food allergy.

### 5.1. Dietary Globalization and Emerging Food Allergens

Globalization has expanded access to foods previously consumed primarily within specific geographic regions, including legumes, seeds, exotic fruits, spices, and concentrated plant proteins [7,8,25,29,30,31,32]. These changes may introduce previously uncommon allergenic proteins into local diets and modify patterns of molecular sensitization and cross-reactivity.

A published case of severe buckwheat-induced anaphylaxis illustrates the potential clinical significance of less familiar food allergens, including those encountered as unexpected ingredients in prepared foods [68]. However, this individual case should be interpreted as a clinical illustration rather than evidence of an increasing prevalence of buckwheat allergy or a causal effect of dietary globalization.

The transition toward plant-based diets has further increased interest in alternative protein sources, including pea, lupine, lentil, chickpea, algae, fungi, and edible insects [8,29,30,31,32,33]. Although these ingredients may contribute to nutritional diversification and food sustainability, their allergenic potential requires appropriate evaluation.

Legume proteins may exhibit structural similarities associated with IgE cross-reactivity, while selected insect proteins, particularly tropomyosin and arginine kinase, may cross-react with allergens from crustaceans and house dust mites [29,33].

However, molecular similarity or positive IgE binding does not establish clinically relevant cross-allergy. The actual risk depends on the protein source, individual sensitization profile, exposure level, and clinical context.

Emerging foods should not be considered intrinsically more allergenic than conventional ingredients. Their clinical relevance must be established through appropriate allergenicity assessment and human evidence.

### 5.2. Food Processing and Allergenicity

Food processing may modify protein structure, stability, digestibility, and IgE-binding properties, potentially influencing allergenic activity [31,63,69].

Thermal treatment, fermentation, enzymatic hydrolysis, and extrusion can alter protein conformation or aggregation. Depending on the allergen and food matrix, these processes may reduce, preserve, or occasionally increase IgE reactivity.

Nevertheless, changes observed in laboratory assays cannot be directly extrapolated to clinical allergenicity. The effects of processing are highly dependent on the specific allergen, processing conditions, and characteristics of the final food product.

This issue is particularly relevant for concentrated plant protein ingredients and alternative protein products, which may undergo processing procedures substantially different from those used for their conventional food sources.

Future investigations should integrate protein characterization, processing-related modifications, immunological testing, and clinically validated outcomes to improve understanding of processing-dependent allergic risk.

### 5.3. Climate Change and Pollen-Associated Food Allergy

Climate change may influence allergenic exposure through alterations in vegetation distribution, pollen production, atmospheric carbon dioxide concentrations, and pollen season duration [26,27,28,70].

These environmental changes are particularly relevant to pollen-associated food allergy, in which primary sensitization to aeroallergens can generate IgE cross-reactivity with structurally related proteins in plant-derived foods [25,34,71,72].

Changes in pollen exposure may therefore modify the geographic context of sensitization and potentially influence the clinical distribution of pollen–food allergy syndrome.

However, direct longitudinal evidence demonstrating that climate change independently increases clinically confirmed food allergy remains limited. Observed associations must be interpreted in the context of geographic variation, dietary habits, aeroallergen sensitization, and diagnostic practices.

Consequently, climate-related influences should be considered plausible modifiers of allergen exposure rather than established independent causes of food allergy.

### 5.4. Allergenicity Assessment and Clinical Surveillance

The increasing use of novel protein sources emphasizes the importance of structured allergenicity assessment and post-market surveillance.

Updated European Food Safety Authority guidance for novel foods addresses the assessment of potential allergenicity, including protein composition, allergen source, sequence similarity, and available experimental evidence [73].

However, current predictive methods have limitations, particularly regarding the identification of de novo sensitization risks and the estimation of clinical reaction severity.

Post-market surveillance may complement pre-market assessment by identifying suspected allergic reactions, characterizing implicated ingredients, and evaluating clinical relevance. Future research should integrate molecular allergology, nutritional exposure assessment, food technology, and standardized clinical outcomes.

Such an approach may support safer food innovation while distinguishing theoretical allergenic potential from clinically meaningful risk. Importantly, these developments should be considered within a life-course framework, as changing dietary and environmental exposures may contribute to different allergic phenotypes during childhood and adulthood.

## 6. Clinical Phenotypes of Food Allergy Across the Lifespan

Food allergy encompasses heterogeneous clinical phenotypes that differ in age at onset, sensitization pathways, molecular targets, and environmental influences. Although childhood represents an important period for allergic sensitization, food allergy may also develop or become clinically apparent during adulthood [2,34,35,36,37]. Adult-onset food allergy, pollen–food allergy syndrome (PFAS), alpha-gal syndrome (AGS), and cofactor-dependent reactions illustrate how nutritional and non-nutritional exposures may influence allergic disease across the lifespan. These phenotypes also emphasize that sensitization, clinical reactivity, and reaction severity are determined by distinct, sometimes interacting mechanisms.

### 6.1. Adult-Onset Food Allergy

Adult-onset food allergy challenges the traditional perception of food allergy as predominantly a childhood disorder. Population-based studies indicate that a substantial proportion of adults with food allergy report developing at least one allergic condition after childhood [38,74].

An important distinction must be made between food allergy that develops for the first time in adulthood and childhood-onset food allergy that persists into adult life. Persistent childhood-onset allergy reflects continued clinical reactivity to allergens encountered earlier in life and should not be interpreted as evidence of sensitization caused by adult-stage exposures. In contrast, adult-onset food allergy may involve newly acquired sensitization, changing environmental or occupational exposures, pollen-associated cross-reactivity, or specific mechanisms such as tick-associated sensitization in alpha-gal syndrome. Nevertheless, the recognition of food allergy in adulthood does not necessarily establish the timing of initial sensitization, particularly when previous reactions were mild, unrecognized, or incompletely documented. Epidemiological studies should therefore distinguish confirmed adult onset from persistent childhood allergy and newly diagnosed disease of uncertain onset. This distinction is essential when interpreting life-course exposure associations and identifying potential opportunities for prevention.

Commonly implicated foods include shellfish, fish, tree nuts, fruits, and vegetables, although allergen profiles vary geographically [2,7,74,75].

Potential contributing factors include new dietary exposures, occupational sensitization, aeroallergen-related cross-reactivity, and changes in epithelial or mucosal environments [9,12,25,34]. However, the causal contribution of dietary transitions or microbiome alterations to adult-onset food allergy remains insufficiently established.

Importantly, adult-onset reactions may reflect distinct sensitization pathways, including primary food allergy, pollen-associated cross-reactivity, or environmentally acquired sensitization. Accurate diagnosis therefore requires careful assessment of dietary and environmental exposure histories, supported by targeted IgE testing and, when appropriate, component-resolved diagnostics or supervised oral food challenges [6,76].

### 6.2. Pollen–Food Allergy Syndrome

PFAS illustrates the interaction between environmental aeroallergen exposure and dietary proteins. Primary sensitization to pollen allergens may induce IgE antibodies that recognize structurally related proteins in plant-derived foods [25,34,71].

PR-10 proteins and profilins are frequently involved and are generally susceptible to heat and digestion, often producing localized oropharyngeal symptoms after consumption of raw fruits or vegetables. Nevertheless, systemic reactions can occur, and clinical severity requires individualized assessment.

Nonspecific lipid transfer proteins (nsLTPs) are comparatively stable and may be associated with systemic plant-food allergic reactions, particularly in Mediterranean populations. However, nsLTP-mediated food allergy should not automatically be classified as conventional PFAS [72].

Regional pollen exposure, dietary habits, and molecular sensitization patterns contribute to geographic differences in clinical expression. Climate-related changes in pollen distribution and seasonality may modify relevant aeroallergen exposures, although direct evidence linking climate change to increasing rates of clinically confirmed PFAS remains limited [26,27,28,70].

### 6.3. Alpha-Gal Syndrome

AGS represents a distinctive environmentally acquired form of IgE-mediated food allergy associated with tick exposure. Sensitization to galactose-α-1,3-galactose may result in allergic reactions following consumption of mammalian meat or selected mammalian-derived products [35,77].

Unlike many conventional food allergies, AGS frequently produces delayed reactions, commonly occurring approximately 2–6 h after ingestion. Clinical manifestations include urticaria, angioedema, gastrointestinal symptoms, and anaphylaxis.

Reaction patterns vary according to the implicated food, amount consumed, and presence of relevant cofactors. Although mammalian meat is the principal dietary concern, selected patients may react to gelatin, dairy products, or other mammalian-derived ingredients [35,77].

Diagnosis combines characteristic clinical history, tick exposure, delayed symptom onset, and alpha-gal-specific IgE testing. Sensitization alone does not establish clinical disease.

AGS demonstrates how a non-dietary environmental exposure can induce clinically relevant reactions to previously tolerated foods, highlighting the importance of exposure-specific mechanisms within the food allergy exposome.

### 6.4. Cofactor-Dependent Food Allergy

Cofactor-dependent food allergy demonstrates that clinical reactivity may depend on the circumstances surrounding food consumption rather than ingestion alone.

Physical exercise, nonsteroidal anti-inflammatory drugs, alcohol, and selected physiological stressors may lower reaction thresholds or amplify allergic responses in susceptible individuals [36,78,79].

Food-dependent exercise-induced anaphylaxis (FDEIA) is a well-characterized phenotype. Wheat-dependent exercise-induced anaphylaxis is particularly relevant and is frequently associated with IgE sensitization to omega-5 gliadin [36,79].

Patients may tolerate the implicated food in the absence of cofactors, complicating diagnosis and contributing to apparently inconsistent reaction patterns.

Clinical evaluation should therefore integrate food consumption, exercise timing, medication use, alcohol exposure, and molecular sensitization profiles. Specialized provocation testing may be considered in selected cases but carries a risk of severe reactions and has imperfect sensitivity.

These observations illustrate that food allergy expression depends not only on allergen characteristics but also on interactions between nutritional exposures and physiological or environmental modifiers.

### 6.5. Clinical and Nutritional Implications

The recognition of distinct food allergy phenotypes has important implications for diagnosis, dietary counseling, and individualized management.

A phenotype-oriented approach should distinguish primary sensitization, cross-reactivity, environmentally acquired allergy, and cofactor-dependent reactions. Positive sensitization tests alone should not justify extensive dietary elimination without appropriate clinical correlation [6,76,80].

Unnecessary avoidance of tolerated foods may compromise nutritional adequacy and dietary diversity, particularly in patients with multiple suspected allergies [80,81,82].

From a life-course perspective, interventions effective for preventing early childhood food allergy cannot automatically be extrapolated to environmentally acquired or cofactor-dependent phenotypes.

Integrating dietary history, molecular diagnostics, environmental exposure assessment, and relevant cofactors may improve clinical interpretation while supporting safe, individualized nutritional management. The major nutritional and environmental exposures, strength of available evidence, and clinical management considerations across different life stages are summarized in Table 3. This comparison highlights the concentration of intervention-supported prevention evidence in infancy and the substantial evidence gaps concerning later-life exposures and adult-onset food allergy.

**Figure 2:**
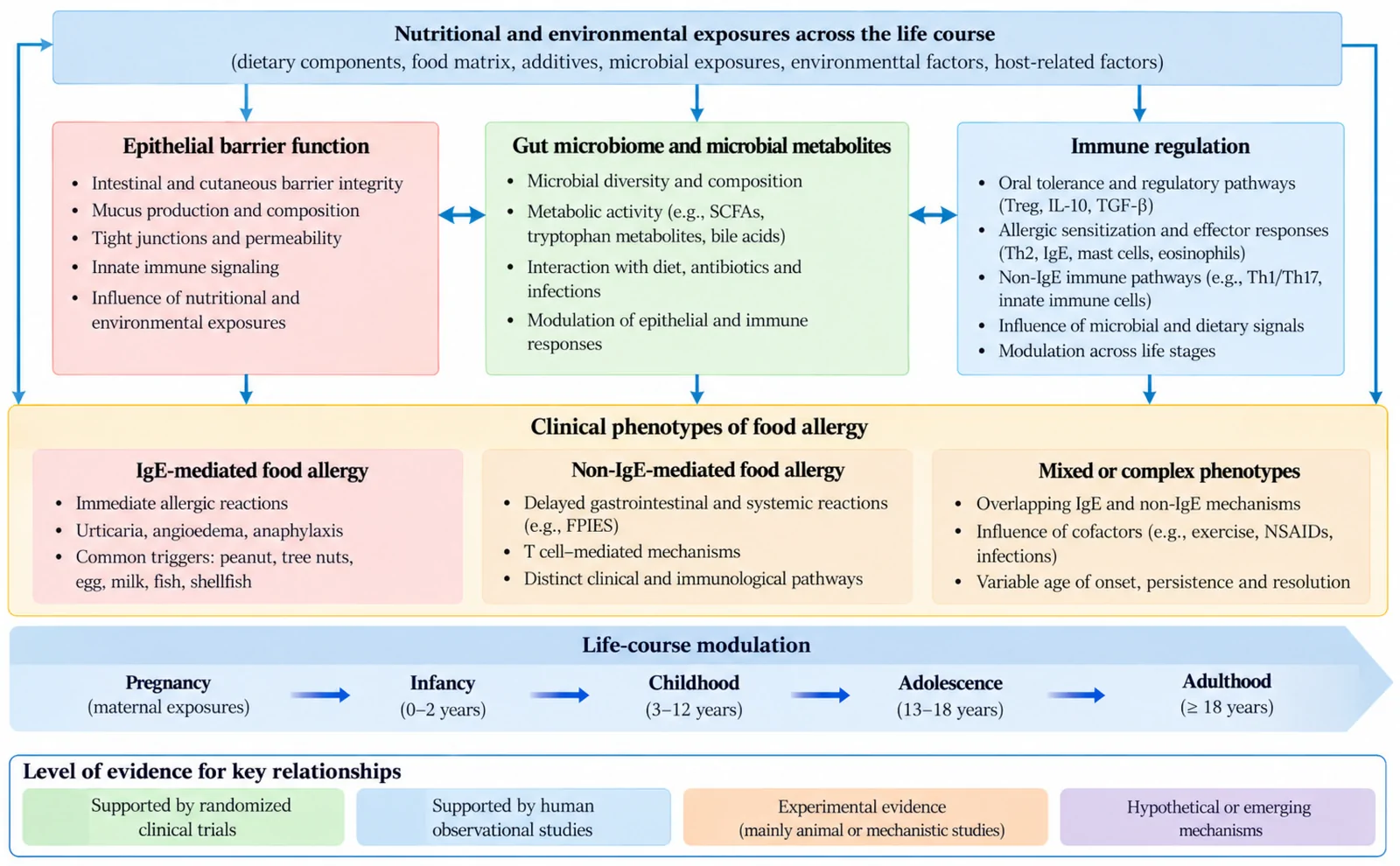
Interactions between nutritional and environmental exposures, epithelial barrier function, gut microbiome activity, and immune regulation across the lifespan. Blue arrows indicate the proposed relationships between exposures, biological pathways, and clinical phenotypes; bidirectional arrows represent interconnected biological processes rather than established causal relationships. The background colors distinguish the main biological components: red/pink represents epithelial barrier function, green represents gut microbiome and microbial metabolites, and blue represents immune regulation. The clinical phenotype categories are highlighted using different background colors for visual differentiation. In the evidence-level legend, green indicates support from randomized clinical trials, blue indicates human observational evidence, orange indicates experimental evidence, and purple indicates hypothetical or emerging mechanisms. The horizontal arrows in the life-course panel indicate progression from pregnancy to adulthood. The resulting clinical phenotypes include IgE-mediated, non-IgE-mediated, and mixed food-allergic responses. Evidence supporting individual pathways ranges from randomized clinical trials to observational studies, experimental models, and mechanistic hypotheses. Changes in immunological biomarkers or sensitization profiles do not necessarily indicate clinically confirmed food allergy. Abbreviations: SCFAs, short-chain fatty acids; Treg, regulatory T cells; IL-10, interleukin-10; TGF-β, transforming growth factor-beta; FPIES, food protein-induced enterocolitis syndrome; NSAIDs, nonsteroidal anti-inflammatory drugs. Abbreviations: SCFAs, short-chain fatty acids; Treg, regulatory T cells; IL-10, interleukin-10; TGF-β, transforming growth factor-beta; FPIES, food protein-induced enterocolitis syndrome; NSAIDs, nonsteroidal anti-inflammatory drugs.

Figure 2 summarizes the principal developmental windows, environmental influences, and emerging clinical phenotypes within the food allergy exposome.

### 6.6. Emerging Allergen-Specific Immunotherapies and Their Relationship to Prevention

Advances in understanding the immunological mechanisms underlying food allergy have stimulated the development of allergen-specific immunotherapies and novel delivery platforms designed to modify established allergic responses. Although these approaches share molecular pathways with the induction of oral tolerance, their clinical objectives differ fundamentally from those of primary prevention. Whereas early dietary allergen introduction aims to reduce the development of food allergy in previously non-allergic individuals, allergen immunotherapy primarily seeks to increase reaction thresholds and achieve desensitization in patients with established food allergy [21,22,23,24,80].

Oral immunotherapy (OIT) involves the controlled administration of gradually increasing doses of a food allergen to promote immunological adaptation. Proposed mechanisms include reduced mast-cell and basophil responsiveness, changes in allergen-specific antibody responses, and modulation of regulatory and type 2 helper T-cell pathways. Current EAACI guidance recommends peanut OIT for children and adolescents with IgE-mediated peanut allergy to achieve desensitization and suggests OIT for selected patients with milk or egg allergy. However, desensitization during treatment should not be equated with sustained unresponsiveness after treatment discontinuation, and treatment-associated allergic reactions remain an important safety consideration. Food immunotherapy should be undertaken by appropriately trained specialist teams [80].

Epicutaneous immunotherapy (EPIT) represents an alternative strategy involving allergen delivery through the skin. The Viaskin Peanut patch uses low-dose allergen exposure through an epicutaneous delivery system, with the aim of promoting immunological modulation while limiting systemic allergen exposure. In the phase 3 EPITOPE trial involving children aged 1–3 years with confirmed peanut allergy, treatment response after 12 months was achieved in 67.0% of participants receiving the peanut patch compared with 33.5% receiving placebo (*p* < 0.001). Anaphylaxis, irrespective of treatment attribution, occurred in 7.8% and 3.4% of participants, respectively. These findings support the capacity of EPIT to increase clinical reaction thresholds, although adverse reactions and uncertainties regarding long-term disease modification remain relevant [83].

Microneedle-based delivery platforms represent another emerging approach. These systems use microscopic projections to facilitate controlled allergen delivery across the superficial skin barrier. Their proposed advantages include improved delivery precision and engagement of cutaneous antigen-presenting cells. In a 2025 experimental study using a biodegradable microneedle array patch containing peanut extract, transdermal allergen-specific immunotherapy reduced anaphylaxis-related responses in sensitized mice, altered allergen-specific antibody responses, reduced Th2-associated inflammation, and increased regulatory T-cell responses. However, these findings were obtained in an animal model and cannot establish clinical effectiveness or safety in humans [84].

Together, these strategies demonstrate the translational importance of allergen exposure route, dose, treatment duration, and immunological context. Nevertheless, prevention of food allergy, desensitization during therapy, and sustained unresponsiveness following therapy represent distinct clinical outcomes and should not be used interchangeably. Emerging allergen-delivery technologies should therefore be considered therapeutic or investigational approaches rather than established methods of primary food allergy prevention [80,83,84].

### 6.7. Diagnosis and Risk Stratification in Food Allergy

Accurate diagnosis of food allergy is essential for appropriate dietary management, avoidance of unnecessary food restrictions, and identification of individuals who may benefit from specialized preventive or therapeutic interventions. Within a nutritional exposome framework, diagnostic evaluation should integrate the clinical history, suspected allergen exposure, sensitization profile, and relevant environmental or physiological cofactors.

#### 6.7.1. Clinical History and Conventional Diagnostic Methods

A detailed allergy-focused history represents the initial step in evaluating suspected food allergy. Relevant information includes the implicated food, quantity consumed, preparation method, interval between ingestion and symptom onset, reproducibility of reactions, clinical manifestations, and potential cofactors such as physical exercise, nonsteroidal anti-inflammatory drugs, or alcohol. The history should also address previously tolerated foods, dietary restrictions, and nutritional consequences [6,76].

Skin prick testing (SPT) and measurement of serum allergen-specific immunoglobulin E (sIgE) are first-line investigations for suspected IgE-mediated food allergy. These methods identify immunological sensitization but cannot independently establish clinical allergy or reliably predict reaction severity. Diagnostic performance varies according to the implicated food, patient population, and pretest probability [6].

In selected circumstances, prick-to-prick testing with fresh foods may improve the evaluation of allergens that are insufficiently represented or altered in commercial extracts. Nevertheless, results require careful interpretation and correlation with the clinical history [6].

#### 6.7.2. Component-Resolved Diagnostics and Molecular Sensitization Profiles

Component-resolved diagnostics (CRD) enable the measurement of IgE antibodies directed against individual allergenic molecules rather than whole-allergen extracts. This approach can improve diagnostic specificity and help distinguish primary food sensitization from clinically less relevant cross-reactivity [6].

For example, IgE sensitization to Ara h 2 in peanut allergy, Cor a 14 in hazelnut allergy, and Ana o 3 in cashew allergy may provide additional diagnostic information when interpreted together with the clinical history and conventional sensitization testing [6].

Molecular diagnostics are particularly relevant in pollen-associated food allergy, in which sensitization to cross-reactive proteins may produce positive extract-based tests without corresponding systemic clinical reactivity. However, component sensitization profiles do not reliably establish individual reaction severity, and molecular testing cannot universally replace oral food challenges [6].

#### 6.7.3. Oral Food Challenges and Clinical Confirmation

The supervised oral food challenge (OFC) remains the reference standard for confirming or excluding food allergy when diagnostic uncertainty persists. It evaluates clinical reactivity under controlled conditions and is especially valuable when the history and sensitization tests provide inconclusive or conflicting information [6].

Open oral food challenges are appropriate in many clinical settings, whereas double-blind, placebo-controlled food challenges may be required in selected circumstances, particularly when subjective symptoms complicate interpretation or in research settings [6].

Despite their diagnostic value, oral food challenges are resource-intensive and carry a risk of allergic reactions, including anaphylaxis. They must therefore be performed in appropriately equipped clinical settings by trained healthcare professionals [6].

Correctly identifying food tolerance is also essential because unnecessary allergen avoidance may reduce dietary diversity, compromise nutritional adequacy, and negatively affect quality of life [80,81,82].

#### 6.7.4. Emerging Functional and Molecular Diagnostic Approaches

Functional cellular assays have emerged as complementary approaches to conventional sensitization testing. The basophil activation test (BAT) evaluates allergen-induced basophil responses using flow-cytometric markers such as CD63 and CD203c. In selected settings, particularly peanut and sesame allergy, BAT may improve diagnostic specificity and reduce the need for oral food challenges [6].

However, BAT availability remains limited, and its performance depends on laboratory expertise, assay standardization, allergen selection, and patient-specific cellular responsiveness. Therefore, BAT should be considered primarily when conventional investigations remain inconclusive, rather than as a universal first-line test [6].

Other investigational approaches include mast-cell activation assays, IgE epitope profiling, and combinations of molecular and functional biomarkers. These methods may provide additional information regarding clinically relevant sensitization and individual reaction thresholds. Nevertheless, further prospective validation is required before they can be incorporated into routine diagnostic pathways [6].

Multivariable diagnostic models integrating clinical characteristics, molecular sensitization, and functional testing may eventually support more individualized risk assessment. Their clinical application requires validation across different age groups, geographic regions, and food allergy phenotypes.

#### 6.7.5. Implications for Prevention and Nutritional Management

Within a life-course nutritional framework, diagnostic evaluation should distinguish individuals with established food allergy from those who are sensitized but clinically tolerant, as well as individuals who have not yet developed food allergy [6].

This distinction is particularly important during infancy, when preventive strategies involving developmentally appropriate allergen introduction may reduce allergy development. In infants with severe eczema or suspected food allergy, individualized assessment may be necessary before introducing specific allergenic foods. However, indiscriminate screening of asymptomatic individuals may produce false-positive results and contribute to unnecessary dietary avoidance [6,21,22,23,24,56].

In patients with established food allergy, accurate diagnosis supports individualized elimination diets, nutritional counseling, reassessment of possible tolerance acquisition, and selection of appropriate therapeutic strategies [6,80,81,82].

Importantly, risk stratification should not be interpreted as evidence that routine screening or biomarker-guided interventions prevent food allergy development. The clinical benefits of diagnostic approaches must be evaluated separately from the efficacy of preventive interventions [6,56].

An integrated approach combining clinical assessment, targeted sensitization testing, selected molecular or functional investigations, and appropriately supervised oral food challenges may improve diagnostic accuracy while supporting safe and nutritionally adequate dietary management [6,76,80,81,82].

## 7. Knowledge Gaps and Future Research Directions

### 7.1. Methodological Challenges and Evidence Gaps

Despite advances in food allergy prevention and nutritional immunology, important uncertainties remain regarding the causal contribution of dietary patterns, microbial metabolites, environmental exposures, and their interactions to clinically confirmed food allergy [9,10,11,12,14,15,16,17,18,19,20].

A major limitation is the heterogeneity of diagnostic definitions. Epidemiological studies frequently rely on self-reported allergy, physician diagnosis, or sensitization testing, whereas oral food challenge-confirmed outcomes are less consistently available [4,5,6]. Since sensitization does not necessarily indicate clinical allergy, standardized diagnostic criteria are essential for evaluating associations between exposures and disease.

Nutritional exposure assessment also presents methodological challenges. Broad classifications such as Westernized dietary patterns or ultra-processed food consumption may obscure differences in nutrient composition, food preparation, processing conditions, and allergenic protein exposure [19,20]. Furthermore, retrospective dietary assessment, confounding, and reverse causality limit interpretation. Dietary restriction may follow suspected food allergy rather than precede its development, potentially generating misleading associations.

Similar limitations affect microbiome research. Although microbial composition and metabolic activity have been associated with allergic outcomes, no reproducible microbiome signature has been validated for routine clinical prediction [14,15,16,17,18]. Most proposed interactions between dietary metabolites and immune regulation remain supported predominantly by experimental or observational evidence.

### 7.2. Priorities for Future Nutritional-Exposome Research

Future research should prioritize longitudinal studies integrating repeated dietary assessment, environmental exposure measurements, functional microbiome profiling, metabolomics, epithelial biomarkers, and standardized food allergy outcomes [10,11,13,14,15,16,17,18].

Prospective investigations extending from infancy into adulthood may help clarify how exposure timing, duration, and cumulative interactions influence the development and clinical expression of food allergy.

Particular attention should be given to microbial metabolic pathways involving short-chain fatty acids, tryptophan-derived metabolites, and bile acid metabolism. However, identifying associations between these pathways and allergic outcomes is insufficient; appropriately designed intervention studies are needed to determine whether modifying them produces clinically meaningful benefits [17,18,62].

Emerging food proteins and changing dietary practices also warrant systematic surveillance. Allergenicity assessment of alternative proteins should integrate molecular characterization, processing effects, immunological findings, and clinical evidence rather than relying exclusively on sequence similarity or IgE binding [31,69,73].

Importantly, future studies should distinguish between food allergy phenotypes. Early-onset food allergy, pollen-associated cross-reactivity, alpha-gal syndrome, and cofactor-dependent reactions involve different sensitization pathways and exposure contexts [34,35,36,74,79].

Geographically diverse cohorts and culturally appropriate nutritional assessments are also needed to improve generalizability and address inequalities in diagnosis, dietary management, and access to preventive interventions [2,5,85,86,87].

Future research should use the nutritional-exposome framework to test causal relationships between exposures and food allergy and to identify potential targets for prevention.

## 8. Conclusions

Food allergy develops and manifests within a changing nutritional and environmental exposome in which dietary exposures, epithelial barrier integrity, microbial ecosystems, and immune regulation interact across the lifespan. However, the causal relevance of these interactions varies substantially among individual determinants.

The strongest evidence for nutritional prevention comes from randomized trials supporting early peanut introduction, with additional but more heterogeneous evidence for egg. In contrast, the proposed contributions of dietary patterns, ultra-processed foods, microbial dysbiosis, and environmental pollutants remain insufficiently established to support specific food allergy prevention recommendations.

The diversification of global food systems and recognition of emerging clinical phenotypes further emphasize the importance of exposure-specific mechanisms and individualized dietary assessment.

Integrating nutritional immunology, environmental exposure research, and clinical phenotyping may improve our understanding of food allergy heterogeneity and help identify modifiable pathways for prevention. Further progress will require robust longitudinal studies, clinically validated outcomes, and careful translation of mechanistic findings into nutritional recommendations.

## Figures and Tables

**Table 1:** Comparative characteristics and outcomes of major randomized food allergy prevention trials.

Trial and Population	Age and Food Intervention	Follow-Up and Diagnosis	Main Findings
LEAP (2015) [21] *N* = 640; infants with severe eczema and/or egg allergy.	4–11 months; peanut-containing foods (~6 g peanut protein/week) vs. avoidance.	To 60 months; peanut allergy assessed mainly by oral food challenge (OFC).	Peanut allergy: 3.2% with consumption vs. 17.2% with avoidance (combined baseline SPT strata).
EAT (2016) [22] *N* = 1303; exclusively breastfed infants from the general population.	From 3 months; peanut, cooked egg, milk, sesame, whitefish and wheat vs. standard introduction (~6 months).	To 1–3 years; food allergy assessed with OFC confirmation.	Intention-to-treat: 5.6% vs. 7.1% (*p* = 0.32; not significant). Per-protocol: 2.4% vs. 7.3% (*p* = 0.01); adherence limits inference.
PETIT (2017) [23] *N* = 147; infants with eczema.	From 6 months; stepwise heated-egg powder (50 mg/day, then 250 mg/day) vs. placebo; active eczema treatment in both arms.	To 12 months; egg allergy assessed by open OFC.	Egg allergy: 8% (5/60) vs. 38% (23/61) in primary analysis (*p* = 0.0001); trial stopped early.
PreventADALL (2022) [55] *N* = 2397; general-population infants; factorial trial.	From 3 months; sequential peanut butter, milk, wheat porridge and scrambled egg (small tastes ≥ 4 days/week); factorial skin-emollient intervention.	To 36 months; clinical assessment with OFC confirmation where indicated.	Food-only vs. no-intervention groups: 0.9% (6/641) vs. 2.3% (14/596). Factorial food-intervention effect: OR 0.4 (95% CI 0.2–0.8); no significant emollient benefit.

**Abbreviations:** SPT, skin-prick test; OFC, oral food challenge; OR, odds ratio; CI, confidence interval. **Note:** Populations, food forms, adherence and diagnostic methods differ across trials. EAT per-protocol results do not supersede intention-to-treat findings. PreventADALL introduction at 3 months should not be interpreted as a general recommendation to introduce complementary foods before 4 months.

**Table 2:** Nutritional exposures and interventions in food allergy: proposed mechanisms, evidence strength, and clinical implications.

Nutritional Exposure/Intervention	Proposed Mechanism	Current Evidence and Clinical Implications
Early peanut introduction	Allergen-specific oral tolerance.	Strong randomized evidence, including long-term follow-up, for peanut allergy prevention in high-risk infants [21,54]. Evidence-based preventive strategy, with age-appropriate introduction and clinical assessment when indicated.
Early egg introduction	Oral tolerance to egg proteins.	Randomized trial evidence supports appropriately timed introduction of well-cooked egg; outcomes differ by preparation, protocol, and population [23,24]. Introduce in an age-appropriate form during complementary feeding.
Dietary diversity	Broader oral antigen exposure and varied microbial substrates.	Observational associations with favorable allergic outcomes; independent prevention of challenge-confirmed food allergy is not established [19]. Encourage a varied, nutritionally adequate diet.
Dietary fiber	Fermentation to short-chain fatty acids (SCFAs); potential barrier and immune regulation.	Mechanistic evidence is substantial, but human interventional evidence for preventing clinically confirmed food allergy is insufficient [17,18]. Support adequate fiber intake for general nutrition; allergy-specific prevention remains unproven.
Ultra-processed foods	Possible effects on microbiota, epithelial function, and inflammatory signaling.	Predominantly observational evidence with heterogeneous allergic outcomes; causality for clinically confirmed food allergy is unestablished [20]. No validated allergy-specific preventive intervention.
Probiotics, prebiotics, and synbiotics	Proposed modulation of microbial composition, metabolism, and immune regulation.	Human intervention results are heterogeneous and strain-/formulation-dependent [56]. Routine use specifically for primary food allergy prevention is not supported.
Vitamin D and omega-3 fatty acids	Potential immunomodulatory effects.	Human preventive evidence is inconsistent or insufficient for clinically confirmed food allergy [56,57]. Routine solely for food allergy prevention is not recommended.
Maternal allergen avoidance	Reduced prenatal exposure to food antigens (hypothesized).	No demonstrated benefit for primary prevention [56,57]. Avoid maternal elimination diets solely to prevent food allergy, unless clinically indicated.

Abbreviations: SCFA, short-chain fatty acid. Note: Evidence is summarized qualitatively rather than by formal GRADE assessment. Experimental mechanisms and observational associations do not establish clinical efficacy. Prevention recommendations should follow current guidelines and individual clinical circumstances.

**Table 3:** Life-course perspectives on food allergy: major exposures, strength of evidence, and clinical implications.

Life Stage	Main Exposures and Evidence	Clinical and Nutritional Implications
Pregnancy and breastfeeding	Maternal diet, allergen exposure, nutritional supplementation, and microbial influences. Evidence is mainly observational; maternal allergen avoidance and routine supplementation have not shown consistent preventive benefits.	Maintain a nutritionally adequate maternal diet. Routine dietary allergen avoidance solely to prevent food allergy in offspring is not recommended.
Infancy	Timing of oral allergen introduction, complementary feeding, eczema, epithelial barrier dysfunction, and microbiome maturation. Strong randomized evidence supports early peanut introduction; evidence for egg is protocol-dependent, and microbiome-directed interventions remain insufficiently validated.	Introduce appropriately prepared allergenic foods during developmentally suitable complementary feeding. Individualize assessment in high-risk infants.
Childhood and adolescence	Continued food exposure, dietary diversity, environmental influences, and persistence or resolution of established allergy. Long-term follow-up supports benefits of early peanut introduction; independent prevention through later dietary modification remains unproven.	Maintain nutritional adequacy, reassess allergy when clinically appropriate, and avoid unnecessary elimination of tolerated foods.
Adulthood	New dietary and occupational exposures, aeroallergen sensitization, tick bites, exercise, medications, and other cofactors. Evidence is predominantly observational and phenotype-specific; primary prevention strategies for adult-onset food allergy remain poorly established.	Distinguish newly acquired allergy from persistent childhood allergy. Assess molecular sensitization, environmental exposures, and cofactors; individualize dietary advice.

Note: The strength of evidence varies across life stages. Randomized evidence for primary prevention is concentrated in infancy, whereas evidence for adult-onset and exposure-dependent food allergy is largely observational. Associations should not be interpreted as proof of causality. Prevention, treatment, and nutritional management of established food allergy are distinct clinical objectives.

## Data Availability

No new data were created or analyzed in this study. Data sharing is not applicable to this article.
